# The etiologies of Kawasaki disease

**DOI:** 10.1172/JCI176938

**Published:** 2024-03-01

**Authors:** Jane C. Burns

**Affiliations:** Kawasaki Disease Research Center, Department of Pediatrics, Rady Children’s Hospital—San Diego, UCSD, La Jolla, California, USA.

## Abstract

Kawasaki disease (KD) is a systemic vasculitis that affects young children and can result in coronary artery aneurysms. The etiology is currently unknown, but new clues from the epidemiology of KD in Japan, the country of highest incidence, are beginning to shed light on what may trigger this acute inflammatory condition. Additional clues from the global changes in KD incidence during the COVID-19 pandemic, coupled with a new birth cohort study from Japan, point to the potential role of person-to-person transmission of an infectious agent. However, the rising incidence of KD in Japan, with coherent waves across the entire country, points to an increasing intensity of exposure that cannot be explained by person-to-person spread. This Review discusses new and historical observations that guide us toward a better understanding of KD etiology and explores hypotheses and interpretations that can provide direction for future investigations. Once the etiology of KD is determined, accurate diagnostic tests will become available, and new, less expensive, and more effective targeted therapies will likely be possible. Clearly, solving the mystery of the etiologies of KD remains a priority for pediatric research.

The cause of Kawasaki disease (KD), the most common cause of acquired heart disease in children, remains a mystery after 50 years of investigation. The historical record suggests that KD was a new disease in Asia after World War II (WWII) and an old disease in the West dating back at least to the 19th century. Where did it come from? Is there one cause or many? Is there a single genetic pattern that confers susceptibility and determines coronary artery outcome? The following is an exploration of answers to these questions.

Diagnosis of KD is hampered by the lack of a specific diagnostic test. Instead, a clinical case definition is used (shown in Table 1) (1). This fact has surely led to both under- and overdiagnosis of KD, problems that would easily be solved if we knew the etiology of KD. Timely diagnosis is imperative, as coronary artery damage from the vasculitis begins early in the course of the illness. A large dose of intravenous immunoglobulin (IVIG) is effective in reducing the prevalence of coronary artery aneurysms from approximately 25% to 5% (2). This therapy, however, is expensive, and the majority of the children who suffer from KD reside in countries where IVIG is not affordable (3, 4). Solving the etiology of KD could lead to more targeted and more affordable therapies so that all the world’s children could be treated. Once the aneurysms form, there is an attendant risk of thrombosis due to altered flow dynamics that include reduced endothelial wall sheer stress and increased particle residence time (5) (Figure 1). For Japanese adults who suffered from KD in childhood and developed giant coronary artery aneurysms, the 10-year cardiovascular event–free survival rate for men was only 52% and for women was 75% (6). For the United States, model predictions indicate that by 2030, 1 in every 1,600 adults will have experienced KD (7).

## Historical perspective

Recognition of a pediatric condition that involved coronary artery vasculitis emerged in Japan in 1970 following a nationwide survey that identified over 3,000 cases and 10 pediatric deaths due to myocardial infarction (8). The survey was initiated following Tomisaku Kawasaki’s 1967 publication describing 50 cases of a mucocutaneous lymph node syndrome that Kawasaki proposed as a new disease (9, 10). Later, historical investigation suggested that KD was new to Japan following WWII (11). A syndrome in infants and children called polyarteritis nodosa had existed in Western countries as far back as the 19th century, with increasing reports of cases in the United States in the early part of the 20th century (12). The pathology of this vasculitis involved a transmural, necrotizing arteritis that was largely limited to the coronary arteries. In 1963, Roberts and Fetterman, two pathologists, described the clinical features of their autopsy cases that included fever, rash, and conjunctival injection, clinical signs that were later associated with KD (13). They proposed that this vasculitis of infants should be renamed periarteritis nodosa of infancy (IPN) to distinguish it from polyarteritis nodosa in adults, a chronic vasculitis that affects arteries throughout the body, most notably in the kidney. In a landmark paper, Landing and Larson reviewed autopsies of KD cases from Japan and IPN cases from the United States (14). They concluded that, pathologically, KD and IPN were the same condition. This raised the question of whether the causative agent of IPN, a rare, fatal condition in infants in the West, was introduced into Japan following WWII. Moreover, it is likely that there were cases of nonfatal KD in the United States that were simply not recognized until after the advent of antibiotic therapy and vaccines that eliminated some of the look-alike rash/fever illnesses in children (12, 15). IPN is no longer diagnosed and is now recognized as a fatal form of KD. However, the claim that IPN and KD are a spectrum of the same disease remains a hypothesis until the mystery of the etiology of KD is solved.

## Epidemiologic observations from Japan provide clues to etiology

**Figure 2:**
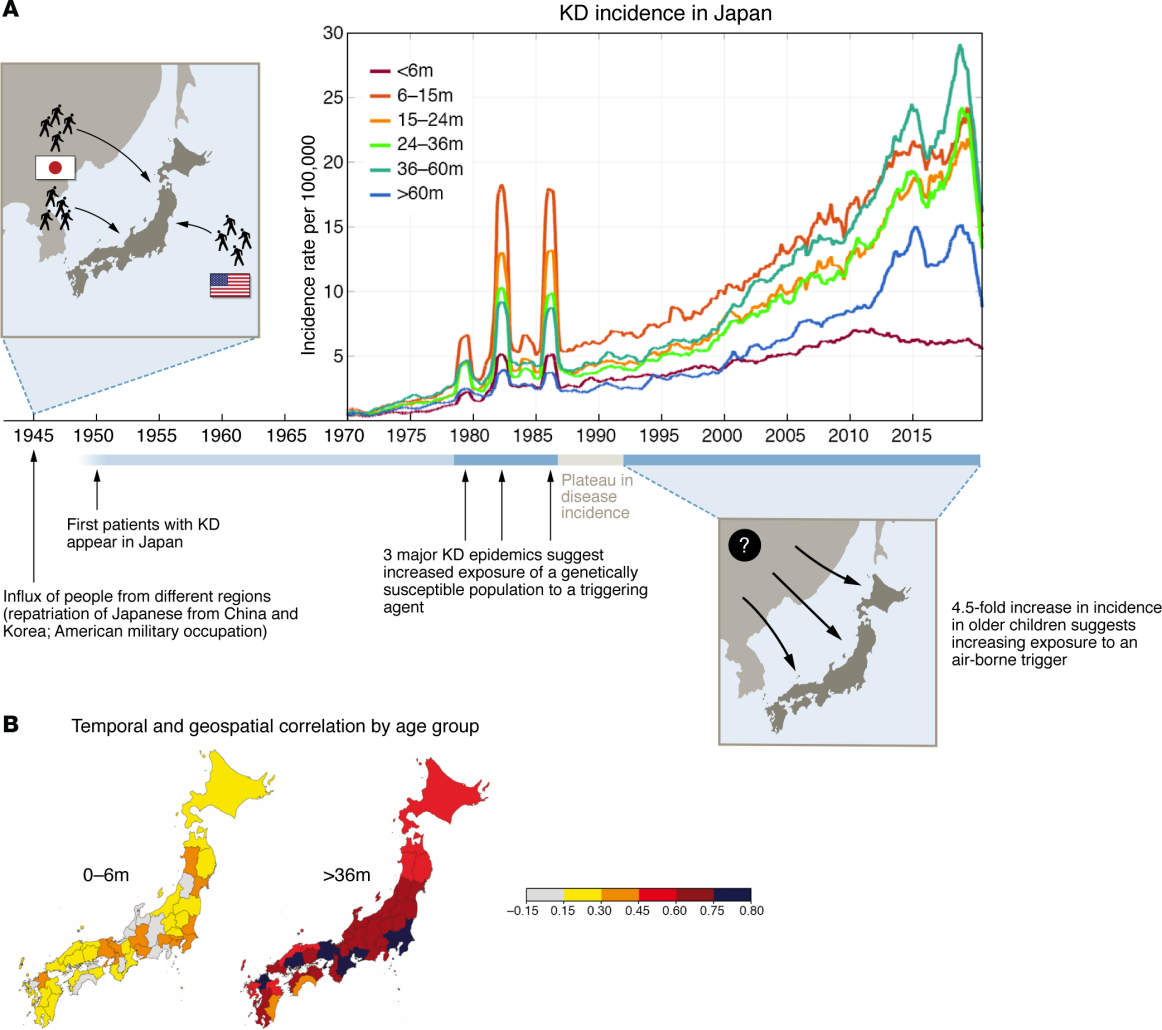
Epidemiologic clues from Japan and the United States. (**A**) Interpretation of epidemiologic clues from Japan’s unparalleled historical record of KD. Left: The first record of KD-like cases in Japan appeared in the 1950s, following large influxes of people from other regions, including repatriation of Japanese individuals from China and Korea as well as US military occupation following WWII and the Korean War. The subsequent 3 epidemics of KD in 1979, 1982, and 1986 are consistent with the introduction of a causative agent into a highly genetically susceptible population. The plateau period from 1988 to 1992 suggests a steady-state exposure to the KD trigger. Right: The 4.5-fold increase in cases in older children occurring from the mid-1990s to 2019 suggests increasing exposure of large populations to an environmental trigger, possibly a wind-borne aerosol. Post-WWII development or increasing intensity of some human activities could create an aerosol that might amplify this exposure, consistent with the rise in KD cases. Incidence in infants remained essentially unchanged over three decades, suggesting a different mode of exposure to the KD trigger in this youngest age group. In 2020, reduced exposure due to isolation measures taken during the COVID-19 pandemic resulted in a decrease in KD incidence that was mirrored by other countries around the globe. (**B**) Correlation of the seasonal pattern of KD across prefectures in Japan, 1988–2019. For infants less than 6 months of age (left) there is little coherence in seasonal patterns across Japan, in contrast to children 3 years and older (right), for whom there is striking synchrony of KD seasonal patterns across all of Japan. KD incidence and correlation data were adapted with permission from *JAMA Network* (16).

The rich epidemiologic record of KD cases maintained by Nakamura and colleagues at Jichi University documented the three nationwide epidemics that occurred in 1979, 1982, and 1986. One cannot escape the interpretation that these massive epidemics were caused by the introduction of a novel agent into a highly susceptible population (Figure 2). Following these nationwide epidemics, there was a largely stable plateau of cases that would suggest a steady state of infections as new susceptible infants entered the population. However, the continued annual rise in cases in school-age children after the mid-1990s argues that a causative exposure is increasing in intensity, with more and more children exposed each year (16). Except for vector-borne diseases, no infectious disease models match the annual increase in KD incidence documented in the Japanese record. An intriguing observation by DeHaan and colleagues is the coherent incidence of KD across broad swaths of Japan, especially in school-age children, which could be consistent with a wind-borne exposure that simultaneously affects a broad geographic region (16). This raised the question of whether a causative agent borne by the wind and coming from a distant location could trigger KD. In contrast to the older children, the infants across all regions in Japan showed virtually no increase in case numbers over the last three decades and no geospatial coherence across prefectures (Figure 2, A and B). These observations suggest either a different mode of exposure to the causative agent or a different trigger for infants, likely encountered in the home.

Many microbes can travel on the wind and cause disease upon arrival at distant locations. As an example of long-range transport of a fungus that retained infectivity, trans-Atlantic airborne transmission of *Aspergillus sydowii* from Africa to the Caribbean caused lethal infection of fan coral (17). Work by Rodo and colleagues suggested that airborne *Candida sp*. coming from northeastern China could be a trigger for KD (18). In an analysis of the greater Toronto region, westerly winds carrying fungal particles were associated with an increase in KD cases (19). Wind-borne bacteria can also travel as aerosols, as evidenced by the association of dust events in the Sahel region in northern Africa, with spikes in cases of meningococcal meningitis (20). Long-range transport of virus particles has been postulated as a mechanism explaining the transmission of both human and avian influenza viruses and their explosive outbreaks over large geographic areas (21, 22). These examples of wind-borne infectious disease transmission highlight the feasibility of long-range transport of a causative agent for KD; however, whether there is in fact a wind-borne trigger remains to be substantiated. Other hypotheses that could explain the annual increase in cases in Japan would be the transmission through aerosols of cofactors that contribute to triggering KD. These could be pollutants causing oxidative stress or heavy metals or toxins attached to aerosol particles that act as cofactors in disease pathogenesis.

In contrast to the large-scale aerosol hypothesis, support for the possibility of person-to-person transmission of an infectious agent also comes from epidemiologic data from Japan. A recent birth cohort study in Japan (>37,000 Japanese children born in May 2010) analyzed KD occurrence in babies aged 6–18 months and identified having an older sibling as a risk factor for developing KD (23). This association was less pronounced if the younger sibling attended day care. Using this same birth cohort, breastfeeding was associated with a reduced risk of KD, while preterm birth was associated with an increased risk of KD, suggesting the possibility of transmission of protective maternal antibodies in the breast milk and across the placenta (24, 25). A sex- and age-matched case-control study in Wuhan, China, and a similar study in Germany with smaller cohorts also found breastfeeding to reduce the risk of KD (26, 27).

## What the COVID-19 pandemic taught us about KD

Although the nature of the KD triggers remains under study, several lines of evidence support person-to-person spread of a causative agent by the respiratory route (Table 2). The most recent natural experiment was the dramatic reduction in KD cases worldwide, which was associated with masking, social distancing, and school closures during the COVID-19 pandemic (28, 29) (Figure 2A). Across the United States, there was a 28% reduction in KD cases across 30 clinical centers, while in San Diego County, where there is active surveillance in place for KD cases, there was a 50% reduction in cases. With the relaxing of masking and return to school, KD resurged to usual numbers. Notably, however, in both the United States and Japan, common respiratory viral infections in children essentially disappeared during the pandemic, while KD did not (30). In fact, the numbers of patients with KD under one year of age did not significantly change from 2020 to 2022 in San Diego County (Figure 2B). This suggests a persistent and prevalent exposure occurring in the home that contributes to infant cases (28). Nonetheless, whatever role person-to-person transmission may play, it cannot explain the more than 4.5-fold increase in case numbers in Japan over recent decades (16) (Figure 2A).

The COVID-19 pandemic had other lessons to teach us about potential triggers for KD. At the start of the pandemic, children with a severe febrile illness that shared some clinical features of KD were reported from northern Italy (31). The new disease was subsequently named multisystem inflammatory syndrome in children (MIS-C) and was characterized by a severe inflammatory response 4 to 6 weeks following exposure to the SARS-CoV-2 virus (32). MIS-C represented a new disease paradigm in which antecedent exposure to an RNA virus could trigger a delayed, systemic inflammatory response. The pathobiology of MIS-C and the mechanisms by which the virus may elicit this response are still under study. However, the idea that a human coronavirus could affect children in this manner led to speculation that perhaps KD, which shared some of the clinical features of MIS-C, might also be a delayed response to an antecedent viral infection. Thus far, antibody profiling of KD sera has failed to reveal a response consistent with known or novel human coronaviruses (33). Nevertheless, the MIS-C disease paradigm is intriguing when considering the potential triggers for KD.

## KD or Kawasaki syndrome

Although KD is commonly regarded as a single disease entity, clinicians have long recognized variability in clinical manifestations and disease outcome among their patients with KD. Using hierarchical clustering on principal components based on 14 variables (age at onset, ten pretreatment laboratory test results, day of illness at the first IVIG infusion, and normalized echocardiographic measures of coronary artery diameters at diagnosis), Wang and colleagues identified four distinct subgroups of 1,016 patients with KD (34). At the molecular level, proteomic analysis suggested subgroup-specific signatures, further supporting the idea that KD may be a syndrome rather than a uniform disease with a single etiologic agent. Whether these subgroups are the result of different triggers or a manifestation of groups of children with slightly different genetic susceptibilities that shape their divergent responses to a single agent remains unresolved. Comparisons of the immune response across these subgroups and genetic analysis within each subgroup have the potential to address this question.

## Clues to etiology from immunologic and microbiologic studies

In the late 1980s, the seminal work of Kappler and Marrack defined a new type of hyperinflammatory state in which a T cell superantigen, usually a microbial peptide, stimulated massive T cell proliferation by cross-linking MHC class II on effector cells to the T cell receptor (35). Subsequently, hypotheses were advanced that the hypercytokinemia in KD was related to a bacterial superantigen (36). However, more detailed microbiologic and immunologic studies failed to support the existence of a superantigen as a driver of the acute inflammatory state in KD (reviewed in ref. 37). Immunologic studies now support the existence of an appropriate immune response to a conventional antigen with the emergence of both CD4^+^ and CD8^+^ memory T cells following the acute illness (38, 39).

Immune profiling of 17 patients with KD prior to treatment revealed different patterns of both innate and adaptive immune response, suggesting that diverse antigens may participate in the pathogenesis of KD (40). Differences in myeloid dendritic cell responses correlated with specific clinical phenotypes, while T cell responses were variable across the cohort. These results suggested the possibility of different antigenic stimuli activating different arms of the efferent immune response in KD. A multicenter transcriptomic and proteomic KD study reached similar conclusions (41). In this study, comparison of whole blood microarray and proteomic data among patients with acute KD or with bacterial or viral infection identified clusters of patients with KD with different patterns. Some KD molecular profiles were similar to those associated with bacterial or viral infection, while some KD profiles resembled neither control group. Similar to the immune profiling study, these findings suggested different antigenic triggers for different groups of patients with KD.

Another approach to understanding the response to the antigenic triggers is to examine the antibody repertoire in patients with acute KD. Using a phage display library expressing 56-residue peptide sequences that span the proteomes of 206 species and more than 1,000 strains of viruses known to infect humans, acute, pretreatment KD sera were profiled and compared with that of age-matched, febrile control children (33). The investigators postulated that even a novel virus belonging to a known genus would share some antigenic determinants derived from highly conserved regions of the genome, such as the polymerase or nonstructural genes. However, there were no differences in antibody profiles between the patients with KD and controls.

Another antibody approach involved cloning antibodies directly from plasmablasts from patients with KD. In 2005, Rowley and colleagues pursued initial observations of intracellular inclusions containing both RNA and protein in KD autopsy tissues that could be consistent with viral inclusion bodies. These structures were stained with synthetic monoclonal antibodies created using cloned immunoglobulin genes from plasmablasts derived from the arterial wall from an autopsy of a patient with KD (42). Although these cytoplasmic inclusions are found in some autopsy tissues from individuals who never had clinical KD, genetically determined response to whatever is causing the inclusions is a critical factor that may influence the development of disease following exposure. Using monoclonal antibodies derived from single plasmablasts from patients with subacute KD, in 2020, Rowley and colleagues showed binding to the bronchial intracytoplasmic inclusions from KD autopsies (43). Using a custom array of known viral epitopes, they identified an epitope derived from a nonstructural protein of the *Hepacivirus* genus that was recognized by monoclonal antibodies derived from clonally expanded plasmablasts from 3 of 11 patients with KD (43). Recently, this group modified the epitope sequence and found that the revised epitope is recognized by monoclonal antibodies derived from plasmablasts isolated from 11 of 12 patients with KD and that these antibodies show convergent VH3-74 heavy chain usage. These data support the idea of a single predominant causative agent in these 12 patients (44). Further work is in progress to develop an ELISA with this modified epitope that could be used to screen larger numbers of patients with KD and perhaps address the KD subgroups described by Wang and colleagues (34).

Several studies have invoked changes in the microbiome as a cause of KD. The majority of studies have been underpowered and made comparisons between patients with KD and healthy controls. All have found differences in the microbiota, which is not surprising, given that inflammation alone can alter the gut microbiome (reviewed in ref. 45). To address the significance of the differences in gut microbiome, adequately powered studies with appropriate matching criteria, including antecedent antibiotic exposure, would need to compare fecal samples between patients with KD and patients with similar levels and duration of inflammation. Even with properly conducted studies, it is hard to reconcile how a change in microbiota could be consistent with the emergence of a new disease in Japan after WWII.

## Contribution of animal models

In the 1980s, Lehman and coworkers described the coronary artery vasculitis that followed intraperitoneal injection of a cell wall extract of *Lactobacillus casei* that they proposed as a murine model for KD (46). These investigators showed that the vasculitis was not related to antibodies directed against the bacterium, nor was there an immune response that targeted the myocardium. The vasculitis could be induced in several different genetic backgrounds but not in C3H/HeJ mice that are known to have a defect in macrophage function. The model has been widely adopted by different groups, with the most detailed studies of disease pathogenesis coming from the Arditi lab where an important contribution was made in demonstrating the central role of the IL-1 pathway (47). Although the model has been elegantly exploited to test different therapies (48), it remains unclear what the model has taught us about etiology. A great contribution would be to apply next-generation liquid chromatography/mass spectrometry to understand the critical component in the bacterial “soup” that is responsible for inducing the vasculitis. This could have important implications for understanding the human disease. A second murine model uses *Candida albicans* cell wall extract, but again, the elements in this complex cell extract that trigger the vasculitis remain unknown (49).

## Environmental factors influence KD susceptibility

Evidence is accumulating that environmental factors may influence or modulate susceptibility to KD. Early studies linked walking or crawling on freshly cleaned carpet with KD, but no causative agent was ever implicated (50, 51). An increased incidence of recent or concomitant respiratory infections in the patient or household members has been noted as a risk factor for KD in several case-control studies (19, 52, 53). Wind patterns and dust transport have been linked to the seasonal variation in KD incidence in such disparate locations as Japan, Chile, and northern Italy (54–56). Environmental pollutants have also been studied in association with KD. No relationship between fine particulate air pollution (PM2.5) and KD was found in a multicenter study in the United States (57). However, studies from Asia have linked KD to ambient ozone levels and exposure to other air pollutants (58, 59). In San Diego, temporal clusters of KD cases were associated with regional-scale air temperature anomalies and consistent larger-scale atmospheric circulation patterns, thus suggesting an environmental exposure that contributes to KD occurrence (60). During the COVID-19 pandemic, regional NO_2_ levels fell as shelter-in-place orders were observed. This created an opportunity to assess the effect of transportation-associated pollution on KD incidence (28). In this natural experiment, census block groups in San Diego that experienced KD cases during 2020 were more likely to have elevated levels of NO_2_, thus suggesting a potential role for oxidative stress in KD susceptibility. Oxidative stress and its contribution to KD pathophysiology has long been a topic of discussion (reviewed in ref. 61). But whether elevated markers of oxidative stress are a cause or a consequence of the inflammatory state in acute KD remains an open question.

## Genetic variation and pathways associated with KD susceptibility

**Figure 3:**
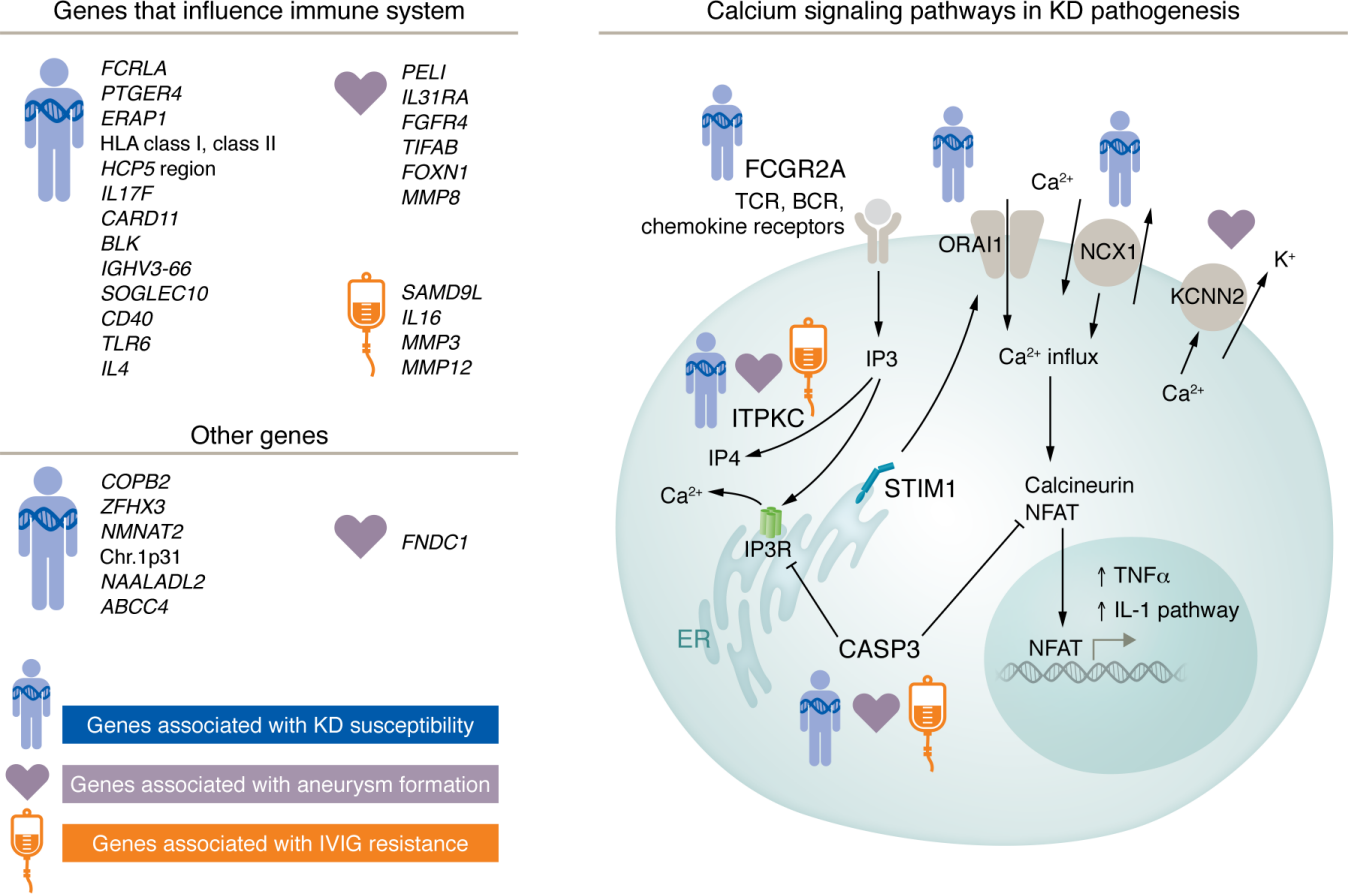
Pathways and genes influencing KD susceptibility and outcome. Figure concept and design are courtesy of Chisato Shimizu.

While there remains debate about potential causative agents and the role of environmental exposures, there is broad consensus that a complex genetic pattern inherited in a non-Mendelian manner shapes susceptibility to KD (62). Several groups have discovered single nucleotide variants of modest effect size that influence KD susceptibility (Figure 3). However, only variants in three genes (*ITPKC*, *CASP3*, and *FCGR2a*) have been validated in independent cohorts across racial and ethnic groups (63–66). Other variants, including a locus in the immunoglobulin heavy chain gene *IGHV3-66*, have only been validated in Asian populations (67). Therefore, the bulk of the literature on KD genetics can be viewed only as hypothesis generating. International collaborations have leveraged the power of multiple cohorts using traditional approaches, including association, family linkage, and GWAS. These efforts taught us that genetic variation that modulates calcium signaling and immune receptor expression influences susceptibility to KD. Japan remains the country of highest incidence, and some variants that affect susceptibility are unique to this population (68). Analysis of pedigrees with multiple affected members has revealed private genetic variations that contribute to susceptibility within that family. In an analysis of whole genome sequence from a family with two affected and two unaffected siblings, the two affected children were compound heterozygotes for two single nucleotide variants in the coding region of Toll-like receptor 6 (*TLR6*) (69). Of interest, the variants mapped to the antigen-combining site of the receptor, suggesting a possible role for TLR6 in KD pathogenesis. These variants, however, were not associated with KD susceptibility when meta-analyzed in independent KD cohorts. These pedigree-based studies with multiple affected members may reveal genetic variation that contributes to susceptibility only within individual families but can be informative regarding biologic pathways involved in disease pathogenesis.

It may be that single genetic variants will not be broadly shared across KD populations but rather influence susceptibility or outcome in specific subgroups of patients. Reanalysis of existing genetic data sets as a function of the clinically defined subgroups of patients described by Wang et al. (30) may prove fruitful. Even though this approach would further reduce sample size, variants associated with KD subgroups might have a larger effect size and may therefore be detectable even in small groups of patients.

One of the many unanswered questions regarding the genetics of KD is whether the propensity to develop coronary artery aneurysms is a genetic trait. Small sample size for this trait that only affects one-quarter of children with KD has traditionally limited the statistical power to address this question. In a multicenter effort, a GWAS of individuals of European descent compared 200 patients with KD with aneurysms to 276 patients with KD without aneurysms (70). The analysis identified an intergenic region on 20q13 with multiple single nucleotide variants meeting genome-wide significance. The most associated variant (rs6017006) was present in 13% of cases and 4% of controls but was absent or rare in East Asian KD data sets. The aneurysm-associated variant did not influence KD susceptibility, thus suggesting that the genetic determinants for coronary artery damage are independent of the variants that influence susceptibility.

Genetic frontiers that have yet to be tackled in KD include analysis of structural variants, including insertions, deletions, and duplications, tandem repeats, and family-based whole genome sequence analysis. Loci that are difficult to analyze using traditional approaches such as the HLA locus on Chr. 6 and the FCγR locus on Chr. 1 will need to be tackled by more advanced statistical methods that may include a graph-based approach that has been successfully used to resolve alleles in the HLA locus using short-read sequencing data (71).

## Conclusions and future directions

Identifying the etiology or etiologies of KD will have a clear effect on diagnosis and treatment and may lead to insights into other “unsolved” conditions such as sarcoidosis and Behcet’s disease that are suspected to have an environmental component. To get across the finish line, we will need increased funding and global, collaborative research initiatives. Once the etiology is solved, specific diagnostic tests will follow, and epidemiology based on confirmed diagnoses will be possible. Current epidemiologic studies surely suffer from both over- and underdiagnosis of KD. It is unknown how often KD is overdiagnosed in children who actually have other rash/fever illnesses or how often the diagnosis is missed, as the illness is naturally self-limited. Data from studies of adult patients with aneurysm and autopsy series from medical examiners suggest that missed KD is a persistent problem whose true magnitude is unknown (72, 73).

**Figure 4:**
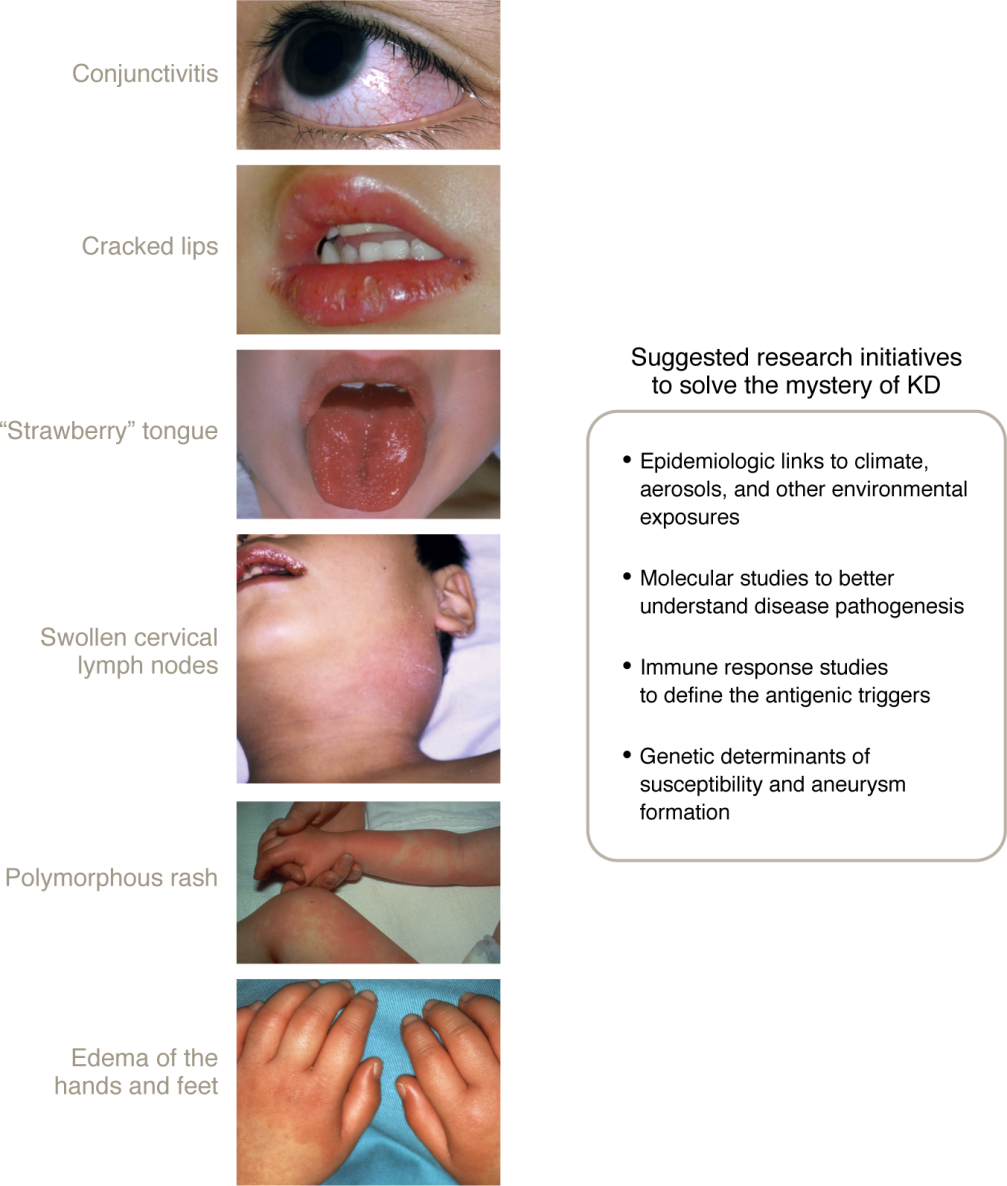
Suggested research initiatives to shed light on KD etiology. The figure also depicts the clinical signs of KD.

KD research is at a crossroads where the decades-long epidemiologic record in Japan can fuel new analytic approaches and novel molecular and machine-learning techniques can now be applied to robust biobanks of KD plasma, RNA, and DNA (Figure 4). Researchers should continue to mine the epidemiologic data from Japan that present two conflicting patterns. On the one hand, these data support the hypothesis of person-to-person spread of a respiratory pathogen. However, the greater than three-fold rise in KD cases in school-age children, coupled with the marked geospatial coherence of KD onsets across Japan, favor exposure to aerosols as the mode of disease transmission. With respect to these hypotheses about a wind-borne causative agent in KD, it remains to be confirmed whether infectious virus can travel on wind-borne aerosols to infect individuals over a large distance or whether there are pollutants borne on the wind that mediate oxidative stress contributing to disease pathogenesis. Analyses of wind trajectories associated with bursts of KD in Japan and elsewhere may help to identify a source region for a wind-borne agent.

Furthermore, the question remains as to whether KD is single disease entity or rather a syndrome with different antigenic triggers. A machine-learning approach revealed that KD might be a syndrome with either different antigenic triggers or genetic variation that leads to different clinical responses to a single agent (34). Research targeting immune response profiling, biomarker discovery, and genetic influences on susceptibility and disease outcome should keep this question in mind. Pursuing the antigenic target of cloned antibodies from plasmablasts derived from patients with acute KD may be a fruitful path. Application of newer molecular tools may also move the field forward. Measurement of cell-free RNA sequences that can be deconvoluted to inform cellular source and therefore tissue of injury may deepen our understanding of disease pathogenesis and potential subtypes of patients with KD. Direct approaches to look for a KD trigger could include metagenomic sequencing of the pharyngeal microbiome or whole blood from patients with acute KD before treatment. Precipitation of antigen/antibody complexes in the subacute phase followed by dissociation and microsequencing of the antigen could be another direct approach.

Research on KD has suffered a funding gap because the exact nature of the disease has not been defined, and, therefore, “ownership” has vacillated among cardiology, infectious disease, rheumatology, and genetics. KD does not fit neatly into autoimmune, infectious, or autoinflammatory categories. Hypotheses regarding KD etiology must explain a minimal set of epidemiologic observations upon which we can all agree (Table 3). After half a century of trying standard approaches to defining KD, it seems likely that understanding this disease will require a paradigm shift. With the application of newer molecular tools and leveraging epidemiologic and genetic big data, the answer may be just around the corner.

## Figures and Tables

**Figure 1:**
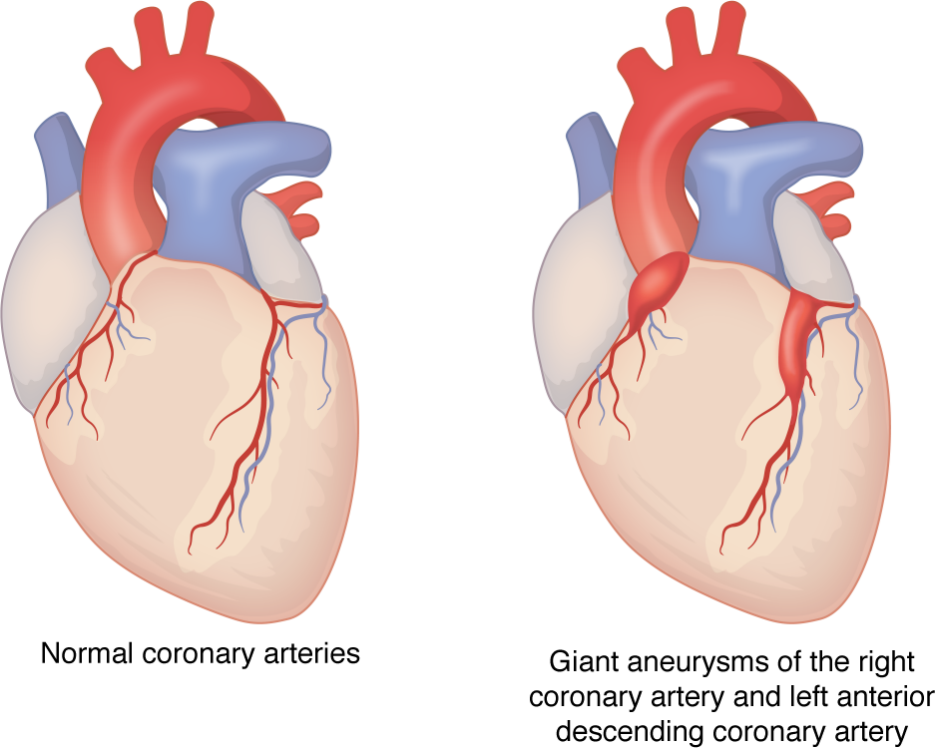
Coronary artery damage from KD. Illustrations represent giant aneurysms of the right and left anterior descending coronary arteries. IVIG therapy can reduce the prevalence of coronary artery aneurysm following acute KD from 25% to 5%, highlighting the need for accurate diagnostic tests and timely treatment.

**Table 3:**
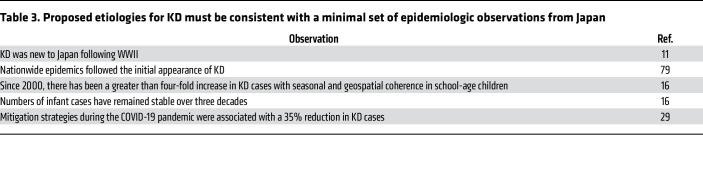
Proposed etiologies for KD must be consistent with a minimal set of epidemiologic observations from Japan

**Table 2:**
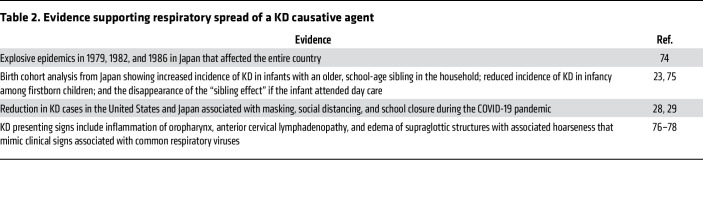
Evidence supporting respiratory spread of a KD causative agent

**Table 1:**
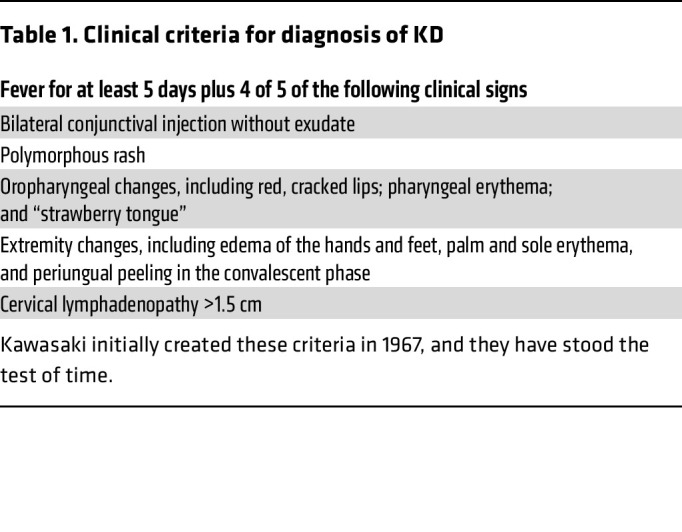
Clinical criteria for diagnosis of KD

## Version 1 03/01/2024

Electronic publication
